# Investigating metabolic activity during oocyte and early embryo development through label-free metabolic imaging: a systematic approach for timelapse applications

**DOI:** 10.1093/humrep/deaf196

**Published:** 2025-11-06

**Authors:** F Horta, A Vuyyuru, H Newman, G Ballerin, S Mercer, E Rolfe, M Haft-Tananian, M Pangestu, P Temple-Smith, B Vollenhoven, R B Gilchrist, S Catt

**Affiliations:** Fertility & Research Centre, Discipline of Women’s Health, School of Clinical Medicine and the Royal Hospital for Women, University of New South Wales, Sydney, Australia; Department of Obstetrics and Gynaecology, Monash University, Melbourne, Australia; City Fertility, Research Support Unit, Sydney, Australia; Fertility & Research Centre, Discipline of Women’s Health, School of Clinical Medicine and the Royal Hospital for Women, University of New South Wales, Sydney, Australia; Department of Obstetrics and Gynaecology, Monash University, Melbourne, Australia; Monash Micro Imaging, The Hudson Institute of Medical Research, Melbourne, Australia; Monash Micro Imaging, The Hudson Institute of Medical Research, Melbourne, Australia; Department of Obstetrics and Gynaecology, Monash University, Melbourne, Australia; Department of Mechanical Aerospace Engineering, Monash University, Melbourne, Australia; Department of Obstetrics and Gynaecology, Monash University, Melbourne, Australia; Department of Obstetrics and Gynaecology, Monash University, Melbourne, Australia; Department of Obstetrics and Gynaecology, Monash University, Melbourne, Australia; Fertility & Research Centre, Discipline of Women’s Health, School of Clinical Medicine and the Royal Hospital for Women, University of New South Wales, Sydney, Australia; Department of Obstetrics and Gynaecology, Monash University, Melbourne, Australia

**Keywords:** oocytes, embryology, metabolic imaging, early development, timelapse-imaging, ART

## Abstract

**STUDY QUESTION:**

Is it possible to assess label-free live cell metabolic imaging during early oocyte and embryo development?

**SUMMARY ANSWER:**

Label-free metabolic imaging can be systematically used during early development, showing no differences between controls and illuminated oocytes and embryos in terms of early development, blastocyst formation, and embryo outgrowth.

**WHAT IS KNOWN ALREADY:**

Non-invasive methods that are reliable to assess oocyte and embryo quality are a significant aim for ARTs. Changes in metabolic activity could lead to cell death or altered early development and low implantation potential. This could potentially be predicted by incorporating non-invasive measurements of metabolism. Metabolic imaging has been investigated through complex methodologies; however, scientific evidence for its utility during early oocyte and embryo development requires further investigation to assess potential translation in clinical settings. Measurements of metabolic activity could be a useful tool, as the autofluorescence of molecules such as nicotinamide adenine dinucleotide phosphate hydrogen (NAD(P)H) and flavin adenine dinucleotide (FAD) are a straightforward representation of mitochondrial function.

**STUDY DESIGN, SIZE, DURATION:**

Female mice (n = 15) and super-ovulated female mice (n = 30) were used to produce oocytes and embryos, respectively. Oocytes and *in-vivo* produced embryos were divided into the control group, sham control group, and illuminated group. Illuminated samples were assessed for both NAD(P)H and FAD levels in oocytes and NAD(P)H levels during early embryo development every 3 h using arbitrary units of autofluorescence (AU). Produced blastocysts were assessed for total cell and inner-cell-mass (ICM) number (by immunostaining for Oct4) and embryo outgrowth assays. Furthermore, safety live birth studies were also conducted.

**PARTICIPANTS/MATERIALS, SETTING, METHODS:**

F1 (C57BL6/CBA) mouse strain was used. NAD(P)H and FAD autofluorescence levels were measured during oocyte and embryo development using confocal microscopy (Olympus FV1200). A confocal Z-stacking function was used to record 15 focal planes, using a 20×/0.95 NA air objective of the entire oocytes and embryos and opening the confocal pinhole system completely. Images were then collected and analysed using FIJI software (version: 2.0.0-rc-69/1.52n; ImageJ). Developmental rates, blastocyst cell numbers, outgrowth rates (for 4 days post blastocyst formation), and live birth rates were assessed.

**MAIN RESULTS AND THE ROLE OF CHANCE:**

Oocyte IVM and embryo culture experiments showed no significant differences in developmental rates between study groups (*P* > 0.05). Similarly, the total number of cells from blastocysts (control: 82.9 ± 5.6; sham: 76.5 ± 3.3; Illuminated: 77.1 ± 4.2; ± SEM) and ICM cells (control: 10.8 ± 1.3; sham: 9.4 ± 0.7; Illuminated: 11.9 ± 0.8; ± SEM) did not differ between groups (*P* > 0.05). Outgrowth assays of the study groups presented similar outgrowth areas during Days 5–8 (post) blastocyst development (*P* > 0.05). Illumination of oocytes demonstrated a significant increase in metabolic activity during IVM, measured by the optical redox ratio (ORR: FAD/NAD(P)H + FAD; *P* < 0.001). Illumination of embryos demonstrated significantly different NAD(P)H activity levels during embryo development, particularly between the two-cell stage (987.1 ± 36.2 AU), morula stage (1226.0 ± 31.5 AU) and blastocyst stage (649 ± 42.9 AU; ± SEM; *P* < 0.05). Additionally, embryos that did not form blastocysts also presented significantly decreased NAD(P)H activity levels at the two-cell stage (normal development: 987.1 ± 36.2; no blastocyst: 726.9 ± 121.7 AU; *P* < 0.05) to the morula stage (normal development: 1226.0 ± 31.5; no blastocyst: 886.0 ± 150.4 AU; *P* < 0.05) when compared with normally developing embryos. Our study indicated that metabolic imaging during early oocyte and embryo development presents no negative effects on developmental rates, blastocyst quality, and embryo outgrowths. Subsequently, live birth rates and offspring health showed no differences between controls and illuminated embryos at the blastocyst stage. Current results provide significant useful information about metabolic activity during live cell imaging as a potential method for timelapse metabolic imaging.

**LARGE SCALE DATA:**

N/A.

**LIMITATIONS, REASONS FOR CAUTION:**

The study was conducted using a mouse model and focused on early oocyte and embryo development, embryo outgrowths, live birth, and early offspring health. Thus, further studies of long-term offspring health are required to fully assess safety and to further validate potential wider applications. Validation in ageing models is also required to assess potential applications for embryo selection.

**WIDER IMPLICATIONS OF THE FINDINGS:**

Measurements of metabolic activity could be applied to determine oocyte and embryo metabolic activity using a variety of microscopy technology with low energy doses as described in this study. Further applications could link the use of metabolic imaging with timelapse technology and artificial intelligence applications to monitor culture conditions.

**STUDY FUNDING/COMPETING INTEREST(S):**

This study was funded in part by a research/educational grant from Ferring Pharmaceuticals, awarded from the Fertility Society of Australia and New Zealand (FSANZ). Funding was also provided in part by the Education Program in Reproduction and Development (EPRD), Department of Obstetrics and Gynaecology, Monash University. F.H. and M.H.-T. have applied for a patent in the topic of metabolic imaging. R.B.G. declares speakers’ fees from Gedeon Richter and Ferring. The other authors have nothing to declare.

## Introduction

Nicotinamide adenine dinucleotide (NAD) and NAD+, including their reduced forms NAD(P)H (nicotinamide adenine dinucleotide phosphate hydrogen) and NADH (nicotinamide adenine dinucleotide hydrogen), alongside cofactors such as flavin adenine dinucleotide (FAD), play essential roles in metabolic pathways involved in cell signalling (Jiang *et al.*, 2011), energy synthesis (Xiao *et al.*, 2018), and DNA repair (Horta *et al.*, 2020, 2021, 2025; Newman *et al.*, 2022), which are in turn impaired with ageing and age-related diseases (Bonkowski and Sinclair, 2016; Horta *et al.*, 2025). During glycolysis and the Krebs cycle, the availability of NAD(P)H/NADH for electron transfer is paramount (Blacker and Duchen, 2016), as these metabolic processes are essential for converting the stored energy of glucose into usable adenosine triphosphate (ATP) (Blacker and Duchen, 2016). Notably, in processes such as oocyte maturation, mitochondria play a pivotal role in supplying ATP through oxidative phosphorylation, with escalating ATP demands during oocyte development (Kang *et al.*, 2024). Similarly, in early embryo development, the Krebs cycle serves as the primary energy provider, but as the embryo progresses towards the blastocyst stage, it increasingly depends on glycolysis (Thompson *et al.*, 2016).

Remarkably, NAD(P)H/NADH and FAD have auto-fluorescent properties, which have been extensively utilized as a label-free technique to monitor the intracellular redox status of living cells and tissues (Chance *et al.*, 1962; Chance and Lieberman, 1978; Blacker and Duchen, 2016; Horta *et al.*, 2025). Because NAD(P)H and NADH have the same spectral peak at 340 nm, their light absorption qualities are identical and their combined autofluorescence signal is labelled as NAD(P)H (Blacker and Duchen, 2016). NAD(P)H, with a peak absorption between the violet and blue wavelengths (Blacker and Duchen, 2016), provides a potentially safer, effective, and viable measure of metabolic activity, as it can be measured outside the violet wavelength range at 405 nm (Chance *et al.*, 1962; Chance and Lieberman, 1978; McLennan *et al.*, 2020) and near infrared spectrum (Blacker *et al.*, 2014).

In order to achieve these measurements, a variety of microscopy methods have been applied to image and quantify NAD(P)H and/or FAD in oocytes and embryos, including confocal microscopy (Wagner *et al.*, 2010; Waldchen *et al.*, 2015; Icha *et al.*, 2017), fluorescent lifetime imaging microscopy (FLIM) (Sanchez *et al.*, 2017, 2018, 2019a,b), hyperspectral microscopy (Rehman *et al.*, 2017; McLennan *et al.*, 2020; Campbell *et al.*, 2022; Tan *et al.*, 2022; Parra *et al.*, 2024), and, recently, light-sheet microscopy (Morizet *et al.*, 2023; Chow *et al.*, 2024; Vargas-Ordaz *et al.*, 2025). This is of relevance as metabolic perturbations of NAD(P)H levels within the oocyte or embryo can be quantified using fluorescence. For instance, levels of NAD(P)H diminish with age in somatic tissue and also decline in immature oocytes (Li *et al.*, 2017; Sanchez *et al.*, 2018; Bertoldo *et al.*, 2020). Similarly, NAD(P)H levels are reduced in oocytes from aged animals when compared with those of young animals, suggesting that reduced NAD+ availability contributes to deteriorating oocyte quality and thus female infertility, which has also been observed via metabolic imaging (Bertoldo *et al.*, 2020; Campbell *et al.*, 2022). Despite this body of knowledge, investigations assessing timelapse metabolic activity in real-time during oocyte and early embryo development require further investigations (Sanchez *et al.*, 2018, 2019a,b), as these are being predominantly focused on specific stages of development rather than investigating the real-time dynamic changes in metabolic activity. Hence, in this study, we present a systematic approach to investigate the autofluorescence of NAD(P)H and FAD in serial time events during oocyte and early embryo development to enable timelapse metabolic activity using specifically defined low doses of energy during oocyte and embryo development. Considering the increasing interest of non-invasive technologies in the field of reproductive biology and ARTs, we identified a lack of systematic methods examining the illumination of energy doses in serial time events, which it could be suitable for measuring autofluorescence of NAD(P)H and FAD in a timelapse setting during live oocyte and early embryo development. Herein, we established a systematic method that is suitable for timelapse investigations in live oocytes and embryos during early development. We hypothesized that this approach is safe for oocytes and embryos, and it has expansion potential to more advanced systems such as multiphoton microscopy, FLIM, light sheet, and hyperspectral microscopy, including future technology innovations in this field.

## Materials and methods

### Study design

A control group was established to assess the standard culture conditions for either oocytes or embryos. In addition, a sham control group was designed to evaluate the viability of oocytes and embryos following handling within a confocal microscope, without exposure to illumination (non-illuminated). A group of illuminated oocytes or embryos was compared to the controls to analyse metabolic activity. Throughout the experiments, all samples underwent incubation under identical conditions to ensure consistency in oocyte and embryo development. For oocytes, metabolic activity assessments were conducted from the germinal vesicle (GV) to the metaphase II (MII) stage, while for embryos, assessments spanned from the two-cell stage to the blastocyst stage. Furthermore, auto-fluorescent measurements were juxtaposed with mitochondrial staining in oocytes, and metabolic activity modulation was implemented during embryo culture. Finally, embryo quality was also assessed to assess whether this illumination approach affects embryo development, outgrowth potential, and live birth rates.

### Experimental animals and ethics

This study utilized female and male F1 Monash Animal Research Platform (MARP) mice (C57BL6/CBA) sourced from the MARP facility in Melbourne, Australia. The mice were maintained under a 12-h light–dark cycle and received attentive care from the personnel at the Monash Medical Centre (MMC) Block B Animal House, situated in Clayton, Melbourne, Australia. Ethical considerations were paramount throughout the study, with all animals euthanized via cervical dislocation for cumulus oocyte complex and embryo collection. The handling, monitoring, and euthanasia procedures adhered strictly to the guidelines outlined in Part 3 of the Prevention of Cruelty to Animals Act 1986 (POCTA) and Part 4 of the Prevention of Cruelty to Animals Regulations 2008 in Victoria, Australia. Ethical clearance for all animal-related protocols was granted by Monash University and the MARP under approval number MMCB 2021/34.

### Metabolic imaging acquisition during oocyte and embryo development

NAD(P)H and FAD autofluorescence levels were measured from the GV to the metaphase II (MII) stage of oocyte development (n = 59). Similarly, NAD(P)H autofluorescence levels were measured from the two-cell embryo stage until the blastocyst stage (n = 35) using confocal microscopy (Olympus FV1200) to compare intensity measurement levels from at least four biological replicates. Single photons from 405 and 488 nm laser sources were used for exciting autofluorescence signal from NAD(P)H and FAD, respectively, using specific and calculated low doses of energy (see section ‘Illumination and light dose settings’). All culture experiments were conducted at 37°C and 6% CO_2_ using a calibrated culture dish (35 mm, ibidi, Germany) with 20 µl drops of culture medium covered by mineral oil. Images were taken at 3-h intervals for a total of 18 h for oocyte development experiments over 66 h to encompass embryo development from the two-cell stage to the blastocyst stage. A confocal Z-stacking function was used to record each entire oocyte and embryo. Images were taken using a 20×/0.95 NA air objective, the pinhole system was opened completely, and images were taken consisting of 15 Z-stack slices with 7 µm between them, following the light dose setting described above. This optimization was applied to avoid photodamage during oocyte and embryo development studies (Horta *et al.*, 2022); however, microscopy settings led to decreased resolution quality compared to standard confocal imaging. Finally, the images were collected and analysed using FIJI software (version: 2.0.0-rc-69/1.52n; ImageJ). Photobleaching experiments using the same microscopy settings were also conducted (Supplementary Fig. S1).

### Illumination and light dose settings

All experiments included a light dose below 50 J·cm^−2^ to avoid cellular damage in live cells (Wagner *et al.*, 2010; Waldchen *et al.*, 2015; Icha *et al.*, 2017). Optimal parameters were set to avoid photodamage using the same excitation wavelengths and acquisition parameters using an Olympus FV1200 confocal microscope to maintain all energy doses below 50 J·cm^−2^.

In order to determine the optimal doses of light across the entire experiments (Supplementary Data Files S1 and S2), the following formulas were applied:


$$A_{lp}=1/4\pi \;{\left(D_{ph}\right)}^{2}$$

where *A_lp_* and *D_ph_* are area of laser spot and pinhole size, respectively. Power density of the laser beam (*P_d_*) for both NAD(P)H (*P_d,N,max_*) and FAD (*P_d,F,max_*) were calculated:


$$P_{d,N,\max}=\mathrm{Laser}\;\mathrm{Power}/A_{lp}$$

$$P_{d,F,\max}=\mathrm{Laser}\;\mathrm{Power}/A_{lp}$$

Considering the maximum laser power used (%) for imaging NAD(P)H and FAD, the delivered power densities (*P_d,N_ and P_d,F_*) to the exposure area were also calculated:


$$P_{d,N}=\%\;\mathrm{Laser}\;\mathrm{Power}\times P_{d,N,\max}$$

$$P_{d,F}=\%\;\mathrm{Laser}\;\mathrm{Power}\times P_{d,F,\max}$$

Then, the total power density (*P_d,T_*) was calculated:


$$P_{d,T}=P_{d,N}+P_{d,F}$$

Conservatively, considering a diameter of an embryo (up to blastocyst stage) as 100 µm, the delivered power density to the imaged embryos was considered proportional to the laser exposure area and can be calculated as follows:


$$P_{d,T,e}=P_{d,T}\times {\left(D_{e}/D_{ph}\right)}^{2}$$

For each sample, 15 Z-stack imaging (*n_img_*) were performed for both NAD(P)H and FAD, and each Z-stack scanning time (*T_scan_*) was 30 s. Therefore, the total energy density delivered (*E_d,T_*) to samples for each biomarker is calculated as follows:


$$E_{d,T}=(P_{d,T,e})\times n_{\mathrm{img}}\times T_{\mathrm{scan}}$$

### Imaging processing

The images were collected and analysed using FIJI software (version: 2.0.0-rc-69/1.52n; ImageJ). Oocytes and embryos were selected as the region of interest (ROI) considering their individual sizes. The same ROIs and area were used to subtract any background noise. Thus, all measurements were normalized to the area of oocyte/embryos. The area, mean, min, and max grey values were obtained. The sum slice’s function was used at every timepoint, selecting the ROI, and the same area was used to obtain the background fluorescence. The area, mean, min, and max grey values were also obtained. An open-source platform, FIJI software (version: 2.0.0-rc-69/1.52n; ImageJ) (Schindelin *et al.*, 2012), was used to visualize the data and to perform the image processing and analyses.

### Metabolic activity measurements

NAD(P)H and FAD autofluorescence levels were calculated by measuring the sum of the intensity at every timepoint. These were also used to calculate the optical redox ratio as the FAD intensity divided by the sum of NAD(P)H and FAD intensity [FAD/NAD(P)H + FAD]. This ratio represents cellular metabolism by measuring the activity of the mitochondrial electron transport chain (Tan *et al.*, 2022). A lower ratio characterizes less active metabolism (Monteiro *et al.*, 2021).

### Cumulus and oocyte collection

Oviducts and ovaries from unstimulated females F1 MARP mice of 4–8 weeks old (Young n = 25) were harvested in warmed gamete handling medium (Gamete buffer, Cook Medical, Bloomington, USA) at 37°C, and GVs were collected by puncturing and gently releasing them from ovarian follicles. The GVs identified were selected irrespective of their morphological condition (with or without cumulus cells) (total GVs; n = 59). Collected oocytes were subject to mechanical cumulus cell removal so that morphology could be assessed during imaging experiments.

### IVM of oocytes

After oocyte collection, GV oocytes were matured in an in-house IVM medium as described previously (Horta *et al.*, 2021) for 18 h at 37°C, 6% CO_2_ in a bench-top incubator (MINC©, Cook Medical). GVs were assessed for maturation status using an inverted microscope (Olympus IX71) at 40× magnification, and oocytes in which the first polar body was extruded were determined to be mature MII oocytes.

### Mice superovulation for embryo culture experiments

Female F1 MARP mice of 4–8 weeks old (n = 20) were stimulated using an intraperitoneal injection of 0.1 ml (5 IU) Pregnant Mare Serum Gonadotrophin (ProSpec, East Brunswick, NJ, USA). Ovulation was induced 48 h later using an intraperitoneal injection of 0.1 ml (5 IU) hCG (Intervet-MSD, Bendigo, VIC, Australia). Female mice were then housed with a stud of proven fertility (n = 10) and assessed for the presence of a vaginal plug 13 h later. All female mice, regardless of mating assessment outcome, were dissected, and their oviducts were harvested at 15–17 h post hCG injection (Intervet-MSD) for embryo collection.

### Embryo collection and culture

Collection of one-cell embryos was achieved by puncturing the ampullary region of the mouse oviducts to release the cumulus–oocyte complexes. The cumulus–oocyte complexes were then transferred to pre-equilibrated culture media (SAGE 1-Step, Cooper Surgical, Fertility Solutions, Denmark; REF 67010060C) at 37°C, 6% CO_2_ in drops of 50 μl covered with mineral oil (Sigma-Aldrich, Missouri, USA; Mineral Oil. Part Number M8410-1L) and cultured overnight in an incubator at 37°C, 6% CO_2_. Then, after 24 h, the resulting two-cell embryos were vitrified using fibre-plug cryo-devices (Cryologic, Blackburn, Australia) and the COOK Blastocyst Vitrification Kit (Cook Medical, US) following kit instructions. The vitrified two-cell embryos were then stored in liquid nitrogen until required for experimentation. Thawing of two-cell embryos was subsequently performed using kit provider instructions (Cook Blastocyst Warming kit, USA). Thawed embryos were randomly divided into the control group (n = 34), sham control group (n = 35), and illuminated groups (n = 35). Then, all embryos were transferred into pre-equilibrated embryo culture media (SAGE 1-Step, Cooper Surgical, Fertility Solutions, Denmark. REF; 67010060C) at 37°C, 6% CO_2_ to continue embryo culture until the blastocyst stage.

### Mitochondrial assessments in oocyte development

Oocytes of different developmental stages were denuded of cumulus cells to obtain a clear signal without excess background during imaging. Oocytes were then transferred to 30 µl microdroplets of 200 nM Mitotracker Red CMXRos (M7512, Thermo Fischer, USA) under mineral oil and kept at 37°C for 30 min. After incubation, oocytes were washed twice in droplets of handling medium for 3 min each. Oocytes were then carefully placed close to each other in droplets of media under oil in a glass-bottomed Ibidi dish for imaging. Experiments were repeated at least four times, and unstained oocytes were used as negative controls. All oocytes were imaged in four channels to facilitate colocalization studies assessed pixel by pixel: brightfield, NAD(P)H, FAD, and Mitotracker.

### Assessment of embryo quality through OCT4 immunocytochemistry

At 4.5 days of culture for all study groups, all embryos were fixed in 3.7% (v/v) paraformaldehyde for 30 min at room temperature (RT) and were then permeabilized in a solution of 0.5% Triton X-100 and 0.1% Sodium Citrate in PBS for 10 min at RT. Embryos were then blocked in 3% BSA/PBS for 1 h at 37°C and incubated with anti-Oct-3/4 Antibody (Santa Cruz Biotechnology, Catalogue #sc 5279) diluted 1:1000 in 1% BSA/PBS for 1 h at 37°C. After primary antibody binding, embryos were washed in 1% BSA/PBS and incubated with Alexa Fluor 568 Goat Anti-mouse conjugated secondary antibody (Abcam Catalogue #ab175701; diluted 1:1000 in 1% BSA/PBS) at 37°C for 1 h. Blastocysts were counterstained with Hoechst 33342 for 5 min (10 µg/ml). Blastocysts were mounted onto Colour frost Microscope Slides (Hurst Scientific) with Vectashield Mounting Medium (Vector Laboratories). The intensity of fluorescent-labelling was assessed against a secondary antibody capturing microscopy images using fluorescence microscopy (Olympus BX43) with all imaging parameters consistent between treatment groups including proper negative controls for each run. Images were collected and analysed in Olympus cell Sens Standard software (version 1.16). The red and blue signal in the nucleus was then used to count all blastocyst cells and inner cell mass cells (OCT4). Additional imaging was conducted using confocal laser scanning microscopy (Olympus FV1200). The confocal Z-stacking function was used to record the dimensions from pole to pole using a 40°ø objective air 0.95 NA of each entire nuclei of blastocyst embryos. Each experiment was conducted on a minimum of five biological replicates, with a minimum of 15 blastocysts analysed. Finally, the images were collected and entered into FIJI software (version: 2.0.0-rc-69/1.52n; ImageJ) to produce microscopic images to the sum of the intensity and Z-stack videos.

### Embryo outgrowths assessment

Blastocyst embryos were transferred after treatment (Day 5) onto 96-well flat-bottomed plates. These plates contained a base layer of 10 μl Dulbecco’s Modified Eagle Medium (Life Technology, Australia) with Matrigel 30% (Corning, Australia). Plates were incubated at 37°C for 30 min to solidify Matrigel, with any excess liquid aspirated and discarded. Embryos were incubated in ORIGIO^®^ Sequential Blast™ media (Cooper Surgical, NSW, Australia) with 1% Fetal Calf Serum (Life Technology, Australia) as outgrowth media. Outgrowth media were discarded and subsequently replaced every 2 days of culture through the outgrowth period. Images of outgrowth were taken either on a Nikon Eclipse Ti2 at 4× magnification at timepoints 48, 72, 90, and 96 h post-embryo transfer. Embryo outgrowth was measured using FIJI software (version: 2.0.0-rc-69/1.52n; ImageJ) by tracing the outgrowth perimeter at least twice per image to obtain the average area in μm^2^ as a measure of outgrowth expansion. Only outgrowth area was used as a measurement of embryo outgrowth, as described by Poh *et al.* (2024).

### Assessment of blastocysts with different metabolic profiles

Embryos were treated without or with FK866 [(E) Daporinad], an inhibitor of nicotinamide phosphoribosyl transferase, NAMPT (Wang *et al.*, 2006; Holen *et al.*, 2008), to assess the effect on the embryo’s metabolic profile. Control samples were two-cell embryos incubated until the blastocyst stage at 37°C and 6% CO_2_ in culture medium (SAGE 1-Step HSA, Cooper Surgical). Under the same culture conditions, the inhibitor group were embryos treated with 30 µM FK866 from the two-cell to blastocyst stage. Three independent experimental replicates were conducted with a minimum of 10 embryos per replicate. Then, the images were collected and entered to FIJI software (version: 2.0.0-rc-69/1.52n; ImageJ). All the intensities for the control and inhibitor samples were compared, and the sum of all the pixel intensity values per image was compared.

### Live birth safety studies

For live birth safety experiments, non-illuminated blastocyst embryos (control group) and illuminated embryos at the blastocyst (illuminated group) were vitrified and thawed as described previously (Vargas-Ordaz *et al.*, 2025) and then transported at 37°C in pre-equilibrated KSOM to the MARP facility. Embryos were transferred into pseudo-pregnant F1 mice following standard operational procedures at MARP, as previously described by Gardner and Sakkas (1993), in groups of 12 embryos (average), with an even number of embryos transferred into each uterine horn. Pregnant mice were then monitored daily by the facility, and at birth, pups were counted and assessed for health clinical observations such as coat, animal activity, breathing, movement/gait, eating, drinking, and alert/sleeping patterns. On Day 7 (D7), Day 14 (D14), and Day 21 (D21) after birth, pups were weighed, sized, and assessed for sex and health clinical observations by MARP staff.

### Statistical analysis

Statistical analyses were performed using GraphPad Prism 10 (GraphPad, San Diego, CA, USA). Data distribution was evaluated using the D’Agostino and Pearson omnibus normality test to derive the mean, SD, and SEM for all parameters. Normally distributed data were analysed using a two-tailed student’s *t*-test or ANOVA with multiple comparisons accounted for using the Bonferroni *post-hoc* method. Non-parametric variables were compared using the Mann–Whitney *U*-test. The Pearson coefficient was used to determine the correlation between NAD(P)H/FAD levels with mitochondrial straining. A *P* value <0.05 was considered as statistically significant in all groups being tested.

## Results

### Metabolic imaging during oocyte maturation: NAD(P)H, FAD mitochondria colocalization

**Figure 1.**
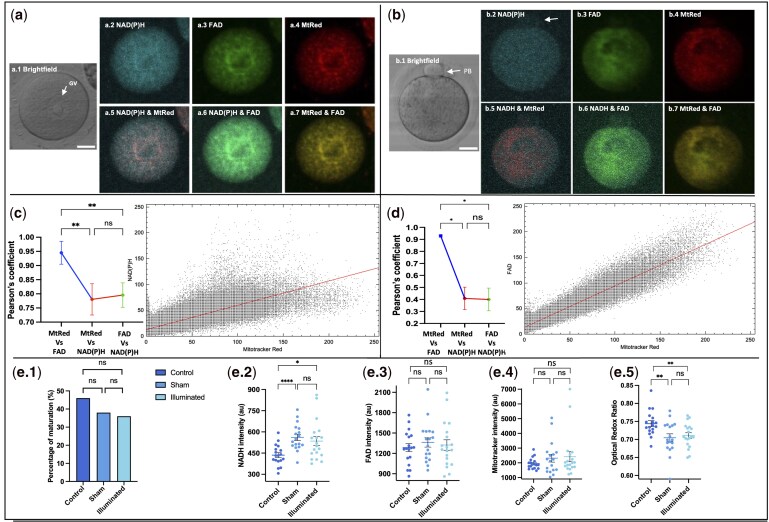
**NAD(P)H and FAD autofluorescence from immature oocytes show high colocalization with Mitotracker Red fluorescence.** (**a**) Mouse germinal vesicle (GV) oocytes were stained with Mitotracker Red (MtRed) and imaged using confocal microscopy. Images (a1–4) show the same GV in brightfield, NAD(P)H, FAD, and Mitotracker channels, and images (a5–7) show merged channels to observe colocalization. (**b**) Mouse MII oocytes were stained with MtRed and imaged using confocal microscopy. Images (b1–4) show the same MII in brightfield, NAD(P)H, FAD and Mitotracker channels, and images (b5–7) show merged channels to observe colocalization. (**c** and **d**) Comparison of fluorescence intensity (arbitrary units) in GV and MII oocytes, respectively, indicated a significant difference (*P*-value < 0.001) between Mitotracker and both NAD(P)H and FAD, including Pearson’s coefficient used to plot differences in pixel colocalization values and cytofluorogram examples. (**e**) IVM rates of oocytes illuminated compared to control and sham controls (e1), including intensity and person comparisons for NAD(P)H, FAD, Mitotracker and Optical Redox ratio (e2–5). Scale bar 20 µm. Data represented as mean ± SEM, while rates as %. One-way ANOVA with Tukey’s multiple comparisons test performed on both intensity and colocalization values. Pearson’s coefficient presented as 95% CIs. Sample size n = 59. Four experimental replicates were conducted. NS, not significant; *P < 0.05; **P < 0.01; ****P < 0.001.

A total of 15 F1 C57BL6/CBA unstimulated females were used. Overall, NAD(P)H and FAD autofluorescence showed known mitochondrial organization at the GV stage as a distinct ring-like perinuclear localization, strikingly similar to that observed in the Mitotracker channel (Fig. 1a). Fluorescence intensity levels from NAD(P)H and FAD were significantly lower compared to that from Mitotracker (Fig. 1c); however, both NAD(P)H (*r* = 0.80 Pearson coefficient) and FAD (*r* = 0.95 Pearson coefficient) demonstrated a high colocalization with Mitotracker at the GV stage (Fig. 1c). In the case of MII oocytes, a uniform dispersed fluorescence was observed in all channels; however, there was a reduced signal from the peripheral cytoplasm in FAD and Mitotracker channels and a weak patch of fluorescence in the polar body of MII oocytes (Fig. 1b). Similar to GV oocytes, MII oocytes showed significantly lower fluorescence intensity levels of NAD(P)H and FAD (Fig. 1d); however, FAD (*r* = 0.95 Pearson coefficient) demonstrated a higher colocalization with Mitotracker than NAD(P)H (*r* = 0.40 Pearson coefficient) at the MII stage (Fig. 1d).

### Oocyte IVM

The effect of illumination was evaluated based on the comparison of IVM maturation rates of the three groups. Post-IVM staining of MII-stage oocytes was conducted to compare NAD(P)H, FAD, ORR, and Mitotracker fluorescence intensity among the groups. The oocyte maturation rates were comparable (Fisher’s exact test: *P* > 0.05) between the study groups (Fig. 1e). There was no effect of illumination on FAD or Mitotracker intensity levels; however, a significantly increased NAD(P)H intensity was observed in the sham (560.9 ± 21.88  arbitrary units of autofluorescence (AU)) and illuminated groups (534.9 ± 31.84 AU), compared to controls (434.8 ± 16.1 AU, *P* < 0.0001; Fig. 1e). However, there was no effect of illumination on any of these measures, compared to the sham controls. GV oocytes showed higher intensity levels for NAD(P)H, FAD and Mitotracker staining compared to MII oocytes. This was reflected by an increased metabolic activity measured by ORR in MII oocytes compared with GV oocytes (Supplementary Fig. S2: *P* < 0.001).

### Metabolic imaging during oocyte IVM

**Figure 2.**
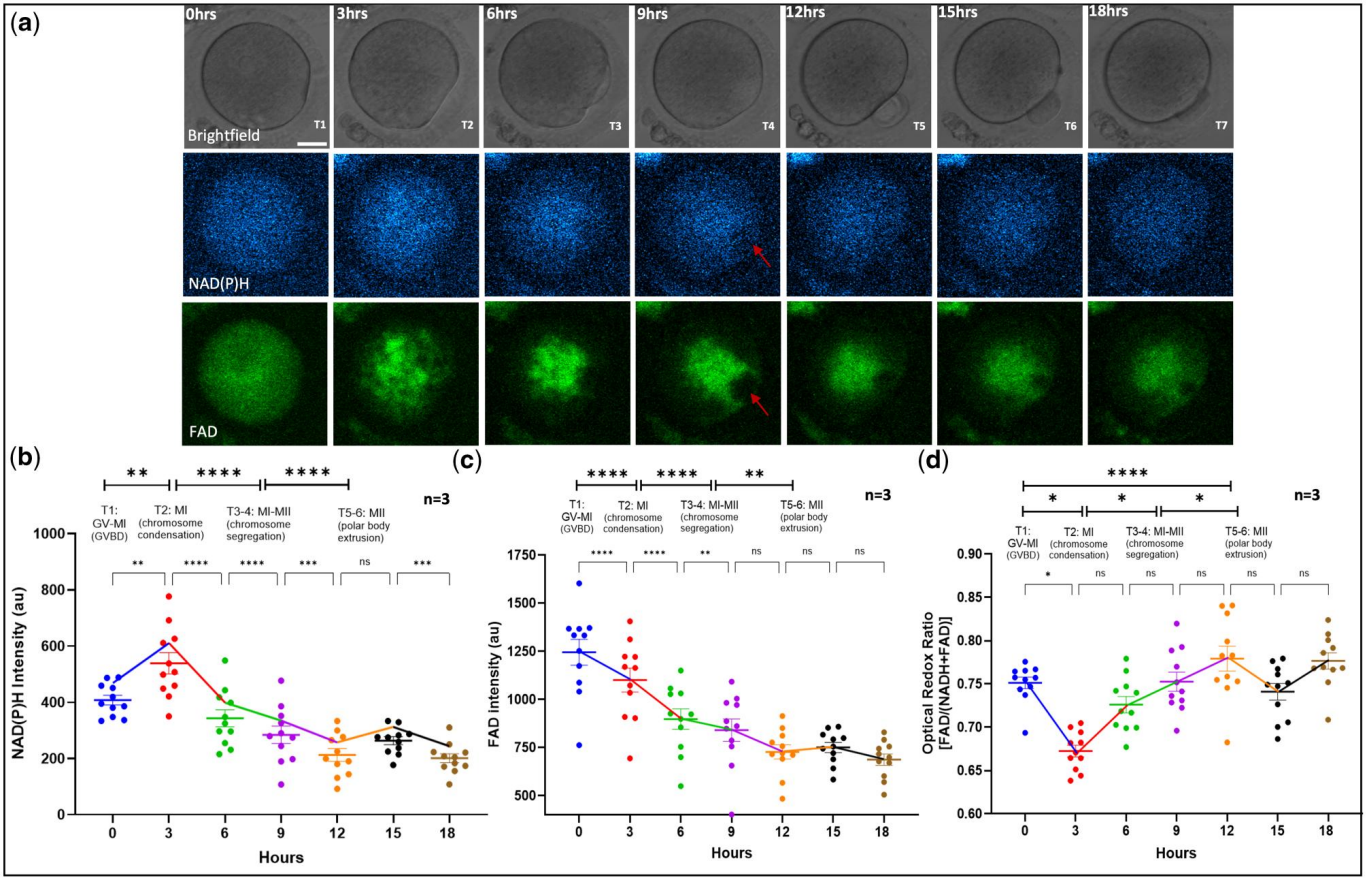
**Changes in metabolic activity during oocyte IVM measured by confocal microscopy.** (**a**) Brightfield, NAD(P)H, and FAD channel images of the same oocyte undergoing IVM over seven timepoints, every 3 h apart. Scale bar 20 µm. FAD autofluorescence visualizes changes in mitochondrial patterns more significantly than NAD(P)H. Time (T) 1 shows the GV beginning to disappear; T2 displays MI chromosomal condensation; followed by spindle formation and chromosome segregation at T3 and T4, the intensity-deficient area of the polar body (red arrow) can be seen surrounded by mitochondria; and finally, at T5 shows MII with the extruded polar body. (**b**–**d**) Time plots of all oocytes that showed normal development. Fluorescence intensity of each oocyte at each timepoint plotted with a line connecting group mean (±SEM) at each timepoint. N, number of biological replicates included; NS, not significant; *P < 0.05; **P < 0.01; ****P < 0.001.

Illuminated oocytes were further analysed during IVM via live metabolic imaging (Fig. 2). Oocytes that matured to MII initially showed a spike in NAD(P)H intensity at t2 (3 h), corresponding with the resumption of meiosis I, characterized by germinal vesicle breakdown (GVBD). Indeed, the ring-like perinuclear organization observed at t1 showed an intense, bright accumulation of auto-fluorescent intensity signals towards the perinuclear region at t2. This accumulation was centrally concentrated, as observed at T3, which was concomitant with a drop in NAD(P)H and FAD intensities. At t4, the central intensity organization showed a spindle-shaped space with a lack of auto-fluorescent intensity as chromosome segregation begins (arrow Fig. 2a), leading to the formation of the first polar body seen at t5 (Fig. 2a). From t5–T7, the auto-fluorescent signals dispersed with their intensity levels gradually decreasing. Overall, a decrease of both NAD(P)H (Supplementary Table S1) and FAD (Supplementary Table S2) was observed through oocyte maturation, indicating a progressive increase in oocyte metabolic activity after GVBD, as measured by ORR (Fig. 2b–d; Supplementary Table S3).

### Metabolic imaging during early embryo development

**Figure 3.**
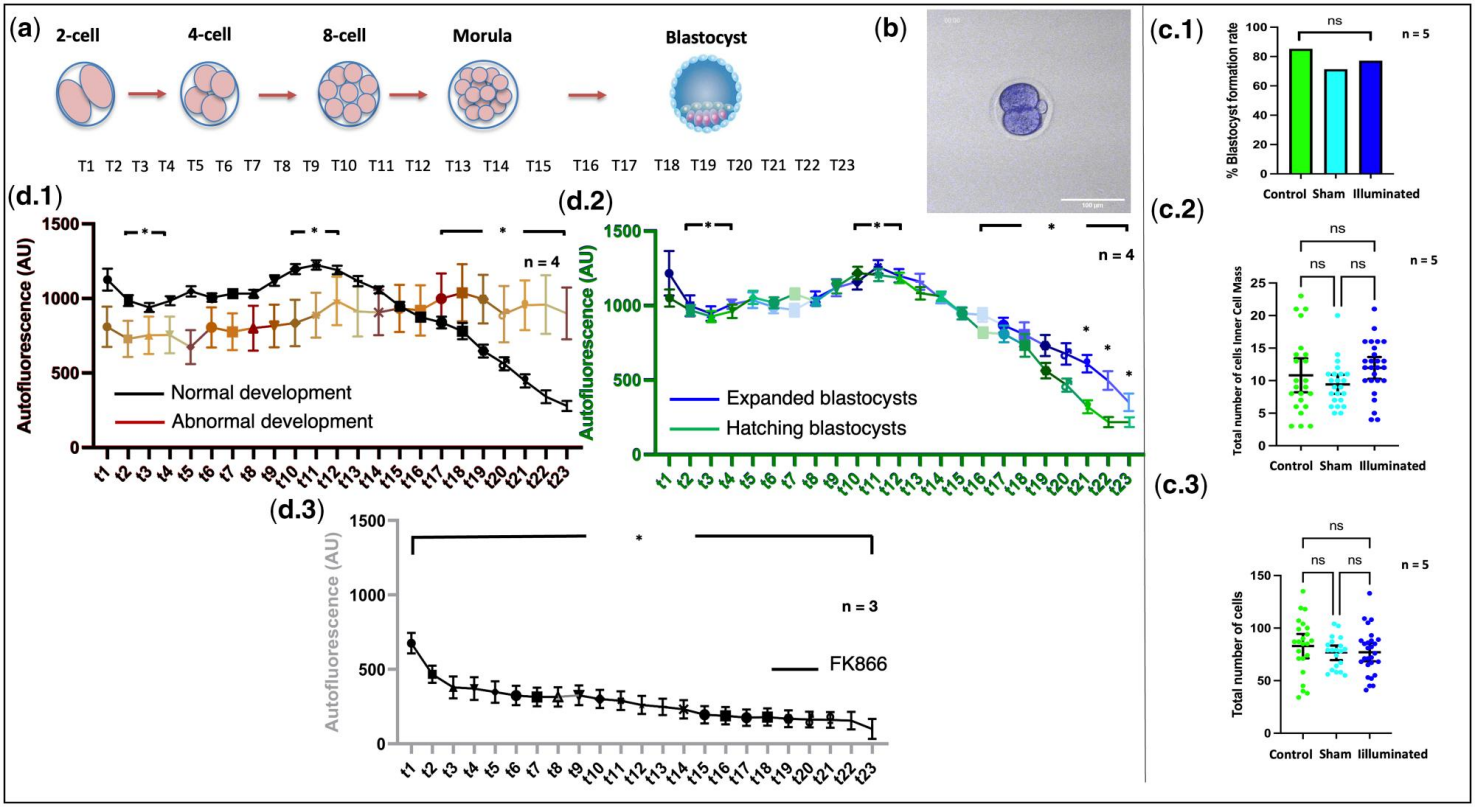
**NAD(P)H intensity levels during early embryo development.** An extensive exploration of NAD(P)H levels was conducted between the two-cell and blastocyst stage (**a**); (**b**: Supplementary Video S1) to examine the NAD(P)H profile during early embryo development. Blastocyst formation rates of illuminated embryos were compared to controls without showing statistical differences (**c.1**; Fisher exact: *P* > 0.05). Similarly, the number of inner cell mass cells (**c.2**) and trophectoderm cells (**c.3**) were also compared to controls without indicated negative effects from the use of illumination (*T*-test: *P* > 0.05). NAD(P)H intensity levels showed differences between major biological stages of embryo development such as the two-cell stage, eight-cell to morula stage, and blastocyst stage (**d.1**), including significantly different NAD(PH) levels in embryos that did not develop to the blastocyst stage (d.1; brown coloured lines: abnormal development; black lines: normal development from two-cell to blastocyst stage). Furthermore, blastocysts that reached the expanded stage (blue coloured lines; **d.2**) and hatching stage (green coloured lines; d2) of development showed similar NAD(P)H levels, but with differences at the time of blastocyst hatching. NAMPT inhibition via FK866 (**d.3**) showed a sharp decline in NAD(P)H intensity levels across all timepoints. Data represented as mean ± SEM while rates as %. NS, not significant; *P < 0.05.

Early embryos were cultured from the two-cell stage (T1) until the blastocyst stage (T23) to perform live cell metabolic imaging during early embryonic development (Fig. 3a; Supplementary Video S1). Illuminated embryos presented significantly different NAD(P)H activity levels during embryo development stages such as the two-cell stage, eight-cell to morula stages, and blastocyst stage (Fig. 3d.1). Embryos that stopped development at any stage but did not reach the blastocyst stage (embryos that failed to form blastocysts) presented a significantly different NAD(P)H activity profile during embryo development compared to those that did reach the blastocyst stage (*P* < 0.05; Table 1; Fig. 3d.1). Indeed, embryos that failed to form blastocysts presented significantly decreased NAD(P)H activity levels at the two-cell stage (normal development: 987.1 ± 36.2; no blastocyst: 726.9 ± 121.7 AU; *P* < 0.05) through to the morula stage (normal development: 1226.0 ± 31.5; no blastocyst: 886.0 ± 150.4 AU; *P* < 0.05), as well as embryo blastocyst stages (Table 1; Fig. 3d.1). In those embryos with normal embryo development, NAD(P)H intensity levels were significantly different between the two-cell stage (987.1 ± 36.2 AU), morula stage (1226.0 ± 31.5 AU), and blastocyst stage (649 ± 42.9 AU; ± SEM; *P* < 0.05; Fig. 3d.1; Table 2). Similarly, normal embryos that fully developed into blastocysts and started the blastocyst hatching process showed differences in NAD(P)H intensity levels at the time of blastocyst hatching compared with those that did not start the hatching process (Fig. 3d.2; Table 3; *P* < 0.01). Furthermore, embryos that were treated with the NAMPT inhibitor FK866 showed a sharp decline in NAD(P)H levels across all timepoints (Fig. 3d.3) compared to those that exhibit normal embryonic development (*P* < 0.05).

**Table 1.** NAD(P)H levels in normal embryo development and embryos that failed to form blastocysts, according to stage of embryo development.

Embryo developmental stage and timelapse timepoint (t)	Normal embryo development (AU ± SEM)	Embryos that failed to form blastocysts (AU ± SEM)	*P* value
**Number (n)**	4	4	−
**Two-cell stage (t2)**	987.1 ± 36.19	726.9 ± 121.7	*0.0294
**Four-cell stage (t3)**	940.4 ± 29.70	751.3 ± 125.5	0.1589
**Eight-cell stage (t7)**	1033.0 ± 30.11	774.8 ± 123.4	*0.0135
**Morula stage (t11)**	1226.0 ± 31.47	886.0 ± 150.4	*0.0065
**Early blastocyst (t15)**	948.9 ± 29.05	931.4 ± 158.4	0.4536
**Blastocyst (t17)**	840.1 ± 39.27	996.9 ± 169.3	*0.0497
**Expanded blastocyst stage (t19)**	649.0 ± 42.86	993.5 ± 163.2	*0.0080
**Hatching blastocyst stage (t21)**	448.4 ± 44.52	952.9 ± 167.0	*0.0080

n, number of biological replicates/animals in study. Values represent mean ± (SEM).

* Statistically different within rows. Mann–Whitney tests were applied. AU, Arbitrary units of autofluorescence.

**Table 2.** Comparison of NAD(P)H levels according to main stages of early embryo development.

	Two-cell stage	Morula	Early blastocyst	Blastocyst	Expanded blastocyst	Hatching blastocyst	*P* value
**Number (n)**	4	4	4	4	4	4	
**Timepoint (t)**	T1	T11	T15	T17	T19	T21	
**AU ± SEM**	1127 ± 73.33^a^	1226 ± 31.47^b^^,^^c^	948.9 ± 29.05^c^^,^^d^^,^^e^	840.1 ± 39.27^c^^,^^d^^,^^g^	649.0 ± 42.86^a^^,^^b^^,^^c^^,^^d^^,^^e^^,^^f^^,^^g^^,^^i^	448.4 ± 44.52^a^^,^^b^^,^^c^^,^^d^^,^^e^^,^^f^^,^^g^^,^^h^^,^^i^^,^^j^^,^^k^	<0.001
**Timepoint (t)**	T2	T12	T16	T18	T20	T23	
**AU ± SEM**	987.1 ± 36.19^b^	1192 ± 28.23^b^^,^^d^	875.7 ± 30.49^c^^,^^d^^,^^f^	782.5 ± 54.22^c^^,^^d^^,^^e^^,^^h^	562.8 ± 43.80^a^^,^^b^^,^^c^^,^^d^^,^^e^^,^^f^^,^^g^^,^^h^^,^^i^^,^^j^	281.0 ± 33.89^a^^,^^b^^,^^c^^,^^d^^,^^e^^,^^f^^,^^g^^,^^h^^,^^i^^,^^j^^,^^k^	<0.001

n, number of biological replicates/animals in study. Values represent mean ± (SEM).

^a,b,c,d,e,f,g,h,i,j,k^Statistically different within rows and columns. ANOVA test and Bonferroni’s multiple comparison test were applied. The two main timepoints of each stage of development were included in the analysis. AU, Arbitrary units of autofluorescence.

**Table 3.** NAD(P)H levels during embryo development according to blastocyst stage of development.

Timelapse timepoints (t)	Normal embryo development (AU ± SEM) Ref	Non-hatching blastocysts (AU ± SEM)	Hatching blastocysts (AU ± SEM)	*P* value
Number (n)	4	4	4	–
**Two-cell stage**				
T1 (0 h)	1127 ± 73.33	1215 ± 149.9	1054 ± 57.46	0.8800
T2 (3 h)	987.1 ± 36.19	996.9 ± 69.49	971.0 ± 57.46	0.9610
**Morula stage**				
T8 (12 h)	1034 ± 25.41	1046 ± 46.99	1029 ± 26.69	0.9806
T9 (15 h)	1121 ± 35.81	1118 ± 57.40	1129 ± 44.78	0.9855
T10 (27 h)	1198 ± 33.71	1158 ± 52.54	1221 ± 41.85	0.6462
T11 (30 h)	1226 ± 31.47	1258 ± 45.52	1208 ± 41.54	0.7282
T12 (33 h)	1192 ± 28.23	1198 ± 44.53	1188 ± 35.40	0.9835
T13 (36 h)	1118 ± 34.39	1158 ± 54.15	1085 ± 41.84	0.5631
T14 (39 h)	1058 ± 23.18	1045 ± 31.81	1065 ± 31.75	0.9102
**Blastocyst stage**				
**Early blastocyst**				
T15 (42 h)	948.9 ± 29.05	946.8 ± 41.83	950.0 ± 38.92	0.9985
T16 (45 h)	875.7 ± 30.49	935.4 ± 46.02	821.5 ± 35.85	0.2913
**Blastocyst**				
T17 (48 h)	840.1 ± 39.27	866.3 ± 50.92	812.1 ± 55.72	0.7833
T18 (51 h)	782.5 ± 54.22	804.3 ± 81.00	735.3 ± 76.42	0.8021
**Expanded blastocyst**				
T19 (54 h)	649.0 ± 42.86	729.9 ± 70.66	565.9 ± 51.78	0.1637
T20 (57 h)	562.8 ± 43.80	673.8 ± 71.85^a^	469.2 ± 44.53^a^	0.0588
**Hatching blastocyst**				
T21 (60 h)	448.4 ± 44.52	608.3 ± 59.19^a^	324.2 ± 43.04^a^	*0.0037
T22 (63 h)	342.6 ± 43.12	495.7 ± 62.88^a^	220.3 ± 34.00^a^	*0.0020
T23 (66 h)	281.0 ± 33.89	349.1 ± 59.09	220.9 ± 32.44	0.1762

n, number of biological replicates/animals in study. Ref, embryos with normal embryo development. Values represent mean ± (SEM).

*^,a^Statistically different within rows. ANOVA test and Bonferroni’s multiple comparison test were applied. AU, Arbitrary units of autofluorescence.

### Safety analysis of illumination approach

**Figure 4.**
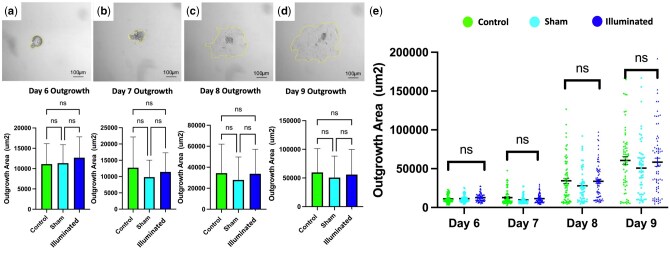
**Embryo outgrowth by area assessment between study groups.** Outgrowth area (μm^2^) was assessed by brightfield (BF) microscopy on (**a**) Day 6, (**b**) Day 7, (**c**) Day 8, and (**d**) Day 9. Images were taken using the Nikon Eclipse Ti2 microscope at 4× magnification with the scale bar representing 100 μm. (**e**) Outgrowth area of blastocysts transferred from different incubation and treatment groups. Outgrowth area assessed by BF microscopy of different treatment groups from Day 6 embryo to Day 9 embryo development. Control (no treatment, N = 80), sham (confocal culturing conditions with no illumination, N = 61), illuminated (adapted confocal microscopy with exposure to illumination, N = 75). Plot points represent values of each individual embryo, and error bars indicate mean ± SEM. NS indicates not statistically significant data. NS, not significant.

In terms of the safety of the measurements during live cell imaging, oocyte IVM studies did not show differences in terms of MII developmental rates between control, sham, and illuminated groups (*P* > 0.05; Fig. 1e). Similarly, embryo culture experiments showed no significant differences in blastocyst formation rates between the study groups (control: 71.7%; sham: 64.9%; illuminated: 71.7%; *P* > 0.05; Fig. 3c). Furthermore, the total number of cells (control: 82.9 ± 5.6; sham: 76.5 ± 3.3; illuminated: 77.1 ± 4.2; ± SEM) and inner-cell-mass (ICM) cells (control: 10.8 ± 1.3; sham: 9.4 ± 0.7; illuminated: 11.9 ± 0.8; ± SEM) did not differ between groups (*P* > 0.05; Fig. 3c). In terms of embryo implantation potential via outgrowth assessment, blastocyst attachment, and outgrowth (trophectoderm expansion) were also measured. The outgrowth parameter for each blastocyst was measured at least twice and therefore averaged to the closest μm^2^. Outgrowth assays demonstrated similar outgrowth areas between treatments, from Days 6 to 9 post-blastocyst development (*P* > 0.05; Fig. 4).

**Figure 5.**
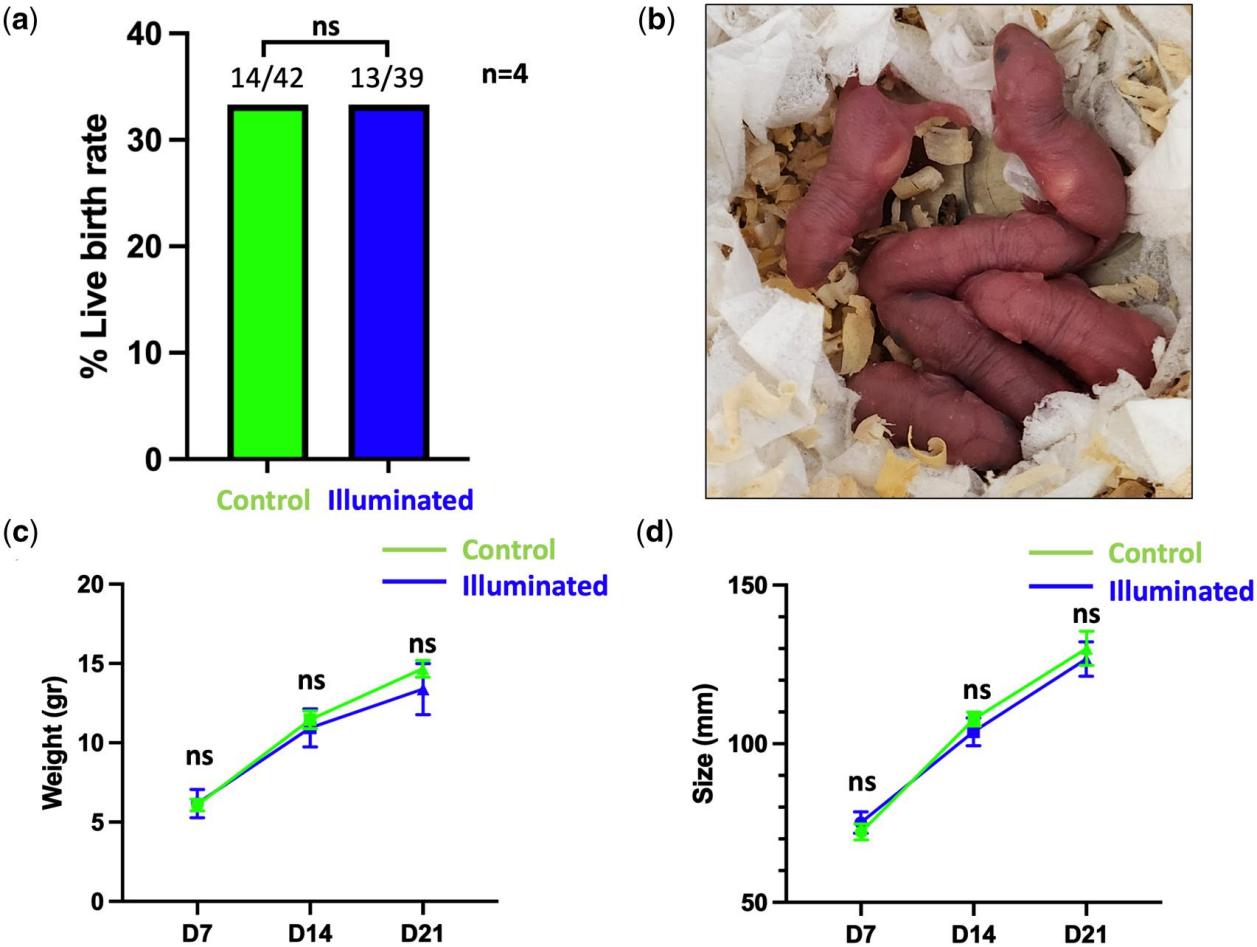
**Live birth rates and health monitoring after illumination in blastocysts.** (**a**) Live birth rate per number of embryos transferred. (**b**) Photo of newborn pups on Day 1 after birth. (**c**) Pups weight health monitoring after birth on Day 7 (D7), Day 14 (D14), and Day 21 (D21). Values represent the mean ± 95% CIs. (**d**) Pups size health monitoring after birth on Day 7 (D7), Day 14 (D14), and Day 21 (D21). Values represent the mean ± 95% CIs. Ns, not significant (Fisher exact test; *P* > 0.05 or ANOVA test with Tukey’s multiple comparison test). n, number of biological replicates.

Live birth safety studies showed no differences for live birth rates between control and illuminated groups (*P* > 0.05; Fig. 5). Similarly, during the first 21 days of development after birth, the health monitoring of pups showed similar weight in grams (control: Day 7: 6.1 ± 0.2; Day 14: 11.5 ± 0.3; Day 21: 14.7 ± 0.2. illuminated: Day 7: 6.2 ± 0.4; Day 14: 10.9 ± 0.6; Day 21: 13.4 ± 0.7; ± SEM; *P* > 0.05; Fig. 5) and size in millimetres (control: Day 7: 72.2 ± 1.2; Day 14: 107.8 ± 1.0; Day 21: 130.1 ± 2.5. illuminated: Day 7: 75.2 ± 1.6; Day 14: 103.8 ± 2.0; Day 21: 126.7 ± 2.5; ± SEM; *P* > 0.05; Fig. 5), including no clinical signs of abnormality during assessments (Table 4).

**Table 4.** Live birth and health monitoring in pups after illumination.

	Live birth rate % (Live births/N embryos transferred)	Females % (n)	Males % (n)
**Control**	33.3 (14/42)	57.1 (8)	42.9 (6)
**Illuminated**	33.3 (13/39)	69.2 (9)	30.8 (4)

	**Day 7**		

	**Clinical observations % (normal)**	**Weight (g)**	**Size (mm)**

**Control**	100	6.1 ± 0.2	72.2 ± 1.2
**Illuminated**	100	6.2 ± 0.4	75.2 ± 1.6

	**Day 14**		

	**Clinical observations % (normal)**	**Weight (g)**	**Size (mm)**

**Control**	100	11.5 ± 0.3	107.8 ± 1.0
**Illuminated**	100	10.9 ± 0.6	103.8 ± 2.0

	**Day 21**		

	**Clinical observations % (normal)**	**Weight (g)**	**Size (mm)**

**Control**	100	14.7 ± 0.2	130.1 ± 2.5
**Illuminated**	100	13.4 ± 0.7	126.7 ± 2.5

n, number of pups. Clinical observations included assessment of coat, animal activity, breathing, movement/gait, eating, drinking and alert/sleeping patterns at birth and Day 7, Day 14, and Day 21 after birth. Values represent mean ± SEM. ANOVA test with Tukey’s test for multi-comparison were applied. No statistical differences were found between control and illuminated groups (*P* > 0.05).

## Discussion

In this study, we explored a label-free metabolic imaging timelapse approach using low-energy illumination doses and autofluorescence that allowed for repeated live-cell assessment of oocytes and embryos during early development. We first investigated how these measurements correlated with mitochondrial staining throughout oocyte development, demonstrating high pixel colocalization values for both NAD(P)H and FAD with the mitochondrial biomarker, Mitotracker. Subsequently, we identified the changes in metabolic activity during oocyte maturation and their correlation with biological milestones, indicating an overall increase of metabolic activity during IVM. In the case of embryos, NAD(P)H dynamics during early development showed remarkable changes between the two-cell stage, morula stage, and the blastocyst stage. Importantly, in both models, we did not observe changes in developmental rates nor changes in oocyte/embryo quality that could be indicative of phototoxicity, suggesting that the use of low energy doses below 50 J·cm^−2^, may be a safe approach to investigate metabolic activity during early oocyte and embryo development.

Oocyte quality affects the oocyte’s developmental competence, defined as its potential to complete meiotic maturation and undergo fertilization, followed by sustaining preimplantation embryo development, implantation, pregnancy, and finally live birth (Conti and Franciosi, 2018). Similarly, mitochondrial distribution has also been directly linked with oocyte quality and the different stages of oocyte development (Bavister and Squirrell, 2000). Thus, investigations using mitochondrial dyes such as Mitotrackers can be relevant to understand the dynamic distribution of metabolic factors such as NAD(P)H and FAD. Mitotracker dyes are innately fluorescent without the need to be oxidized or reduced to emit fluorescence (Poot *et al.*, 1996). They use the highly negative mitochondrial membrane potential to enter the mitochondria, allowing the dye to be retained independent of mitochondrial electrical potential (Dumollard *et al.*, 2007; Kholmukhamedov *et al.*, 2013). Thus, its localization in the mitochondria allows accurate visualization of mitochondrial distribution, and, depending on the dye used, its fluorescence intensity reflects changes in mitochondrial function (Poot *et al.*, 1996). Hence, comparing mitochondrial location and activity using Mitotracker in GV, MI and MII oocytes against metabolic imaging for NAD(P)H and FAD by autofluorescence in the same oocyte enabled us to observe a strong positive correlation between NAD(P)H and FAD autofluorescence with mitochondrial labelling. The dynamic changes of oocyte meiotic maturation were reflected in variations in NAD(P)H and FAD intensity levels. The optical redox ratio, representing metabolic activity, also reflected these changes, with increasing levels during oocyte maturation. Indeed, oocyte maturation has been generally characterized by increased mitochondrial activity and ATP production to facilitate the transition from resumption of meiosis to MII arrest (Dalton *et al.*, 2014). This increase in activity has been linked with higher ATP demands for this active phase of nuclear maturation: MI oocytes involving chromosomal condensation and spindle migration within the oocyte, and MI transitioning to MII requiring chromosome segregation and extrusion of a polar body (Kang *et al.*, 2024). A strong correlation between mitochondrial distribution patterns and increased ATP production has been found with oocyte development during oocyte maturation (Yu *et al.*, 2010). These concepts of increased metabolic activity with oocyte maturation are consistent with the patterns we observed using real time, repeat-measures in live oocytes.

In terms of early embryo development, our findings revealed that NAD(P)H levels gradually increased from the two-cell stage to reach a maximum at the morula stage and subsequently declined in the blastocyst stages. Similarly, studies using FLIM technology have shown a similar NAD(P)H profile during mouse embryo development (Sanchez *et al.*, 2019a). Considering these findings, we hypothesize that the decrease in NAD(P)H levels was primarily due to metabolic shifts associated with increased glycolysis (Thompson *et al.*, 2016). This suggests that higher metabolic activity (Sanchez *et al.*, 2018; Seidler *et al.*, 2020) may necessitate higher levels of NADH transported into mitochondria to donate electrons for energy production. Furthermore, we observed that embryos with normal development rates, exhibited different metabolic profiles when compared to embryos that failed to form blastocyst, or slower-developing embryos that did not reach the blastocyst stage. Similarly, it has been shown that NAD(P)H intensity was altered in cases of embryo developmental delays due to hypoxia-induced damage to embryos (Seidler *et al.*, 2020). Moreover, it has been also found that embryos can respond to damage or stress, showing delays in embryo development to combat associated damaging stress (Casanovas *et al.*, 2019; Newman *et al.*, 2022). This suggests that there could be a link between NAD(P)H metabolic activity and developmental delays from stress-induced damage in embryos. On the other hand, we inhibited NAMPT activity using FK866, which blocks the salvage pathway of NAD+ biosynthesis (Hasmann and Schemainda, 2003). This inhibition led to a significant decline in NAD(P)H levels during embryonic development. As a result of NAMPT inhibition, FK866 induces a progressive depletion of NAD+ and a simultaneous depletion of NAD(P)H in human cell lines (Hasmann and Schemainda, 2003) as we also observed during embryonic development, indicating that the fluorescence detected in the embryos was NAD(P)H autofluorescence.

Nevertheless, the approach of this study presents some limitations. NAD(P)H and FAD are not fluorophores but molecules which are auto-fluorescent, whose signal intensity has been linked to the health and metabolic state of the oocytes and embryos (Horta *et al.*, 2025). Thus, this method presents limited potential for investigations of photobleaching of NAD(P)H and FAD, which is typically associated with fluorophores by a decline in fluorescence, often followed by recovery of fluorescence (Icha *et al.*, 2017). For this reason, a focus on identifying noneligible phototoxicity was established as a key validation point (Icha *et al.*, 2017). Furthermore, we used a 405 nm laser, which lies at the boundary of the UV and visible light spectrum, providing only a fraction of NAD(P)H autofluorescence due to the peak excitation range for NAD(P)H being between 320 and 380 nm (Blacker and Duchen, 2016). However, using shorter wavelength radiation (340–400 nm) in the UVA region has been shown to cause oxidative stress, release of reactive oxygen species, and damage to biological components (Lawrence *et al.*, 2018). Therefore, using a UV laser would likely be harmful to the oocyte and embryo, impeding potential application in a clinical ART setting. Importantly, in this study, we opened the confocal pinhole, which served to better distinguish NAD(P)H intensity measurements but reduced the resolution power and increased the signal-to-noise ratio of images. Despite this, the NAD(P)H signals detected correlated with biological milestones in both oocytes and embryos during early development and decreased signals during NAMPT inhibition, indicating that biologically meaningful information was able to be obtained. Furthermore, it is important to recognize that our IVM rates were not as high as they could be, especially considering the significant improvements in current IVM culture systems (Gilchrist and Smitz, 2023) and the methodology we employed. Indeed, although our study groups did not present statistical differences between them, there was a tendency for reduced IVM rates in both sham and illuminated groups (8–10%). This could be attributed to a suboptimal confocal culture environment that may require further refinement to minimize potential adverse environmental impacts on oocytes. Furthermore, we also included oocytes with and without cumulus cells from all groups to assess the correlation between oocyte morphology, mitochondrial staining, and metabolic imaging, without separately assessing denuded and cumulus-enclosed oocytes. Indeed, this is a limitation, as cumulus cells are known to play a significant role during IVM (Gilchrist *et al.*, 2008), so this could explain tempered IVM rates and influence the range of NAD(P)H/FAD intensities. In addition, the IVM culture system could have been further improved by the introduction of a pre-maturation step to facilitate synchronization of oocyte maturation and subsequent performance of oocyte developmental potential (Gilchrist *et al.*, 2024). Hence, future studies could also include an independent analysis of denuded and cumulus-enclosed oocytes to further assess their impact on IVM rates, as well as advanced microscopy technologies such as light sheet (Vargas-Ordaz *et al.*, 2021, 2025; Morizet *et al.*, 2023; Chow *et al.*, 2024). These systems could allow independent 3D analysis of the oocytes and surrounding cumulus cells.

The knowledge provided in this study could inform the use of alternative advanced technologies such as FLIM (Sanchez *et al.*, 2019a; Shah *et al.*, 2022; Venturas *et al.*, 2022; Sakkas *et al.*, 2024), hyperspectral microscopy (Tan *et al.*, 2022; Parra *et al.*, 2024), and light-sheet microscopy (Vargas-Ordaz *et al.*, 2021; Morizet *et al.*, 2023; Chow *et al.*, 2024; Vargas-Ordaz *et al.*, 2025) as a potential approach to perform timelapse metabolic imaging assessments during oocyte and early embryo development. In contrast with this study, current studies using advanced microscopy technologies are mainly restricted to specific timepoint investigations during development rather than label-free imaging during early development (Horta *et al.*, 2025). Nevertheless, the use of light-sheet microscopy could facilitate label-free imaging with a higher number of images by an increased axial sampling, Z axis, as the small depth of focus used during illumination leads to a high number of images (70 to >100) obtained from short periods of illumination (Morizet *et al.*, 2023; Chow *et al.*, 2024; Vargas-Ordaz *et al.*, 2025). This stands to provide high 3D image quality, as it increases the number of focal planes for data analysis compared to previous studies using technologies such as FLIM (Sakkas *et al.*, 2024) and hyperspectral (Tan *et al.*, 2022; Parra *et al.*, 2024). Despite this, studies using advanced microscopy systems (Tan *et al.*, 2022; Parra *et al.*, 2024; Sakkas *et al.*, 2024) have been able to show clear links between metabolic imaging and biological insights, such as metabolism (Parra *et al.*, 2024) and aneuploidy status (Tan *et al.*, 2022; Sakkas *et al.*, 2024). These are significant points that could be further investigated with the approach presented in this study. Similarly, as described in Vargas-Ordaz *et al.* (2025), we also tested metabolic inhibition with FK866, but analysing the dynamics of NAD(P)H during embryo development showing a profound decrease of NAD(P)H after 3 h of embryo culture. Considering this, future studies employing timelapse metabolic imaging could assess the effect of novel interventions *in vitro*, such as the use of nicotinamide mononucleotide (Bertoldo *et al.*, 2020) and the use of antioxidants (Truong and Gardner, 2017) during IVM and/or embryo culture. This could be of potential benefit, as current culture systems are static, impeding the removal of interventional compounds under investigation during culture. Thus, analysing the dynamics of metabolic activity could facilitate monitoring treatments *in vitro* during specific stages of development and/or personalizing interventions according to the metabolic state of oocytes and embryos.

In terms of the safety of the illumination doses, we did not observe differences between controls, sham, and illuminated samples on either oocyte or embryo developmental rates. Similarly, we did not observe differences in embryo quality at the blastocyst stage by determining ICM and trophectoderm cells, as well as assessments of embryo outgrowths. A limitation of the study is that the *in vitro* embryo outgrowth experiment is a poor measure of embryo implantation potential (Poh *et al.*, 2024). Thus, future studies could perform additional assessments to assess the embryo implantation potential, such as epiblast expansion (Weberling and Zernicka-Goetz, 2021). This would be useful to better understand the physiological status and mechanisms of embryo implantation, which was not the focus our study. Current results suggest that repeated low doses of illumination during oocyte and embryo development may be safe, as embryo transfer studies showed no differences in terms of live birth rates and offspring health during the first 21 days of growth compared to controls. Nevertheless, before any clinical application could be contemplated with similar light doses, further safety studies such as lineage patterning (Plachta *et al.*, 2011) and DNA damage on embryos (Chow *et al.*, 2024) are warranted to exclude the possibility of DNA damage, mutations, and/or long-term developmental effects on the embryo or offspring health.

## Conclusion

In this study, we developed a systematic approach to conduct serial measurements of timelapse metabolic imaging in live oocytes and embryos during early development. The approach was label-free, and we were not able to detect any adverse effects of illumination on oocyte and embryo viability, outgrowth potential, live birth rates, or offspring health, although future studies should consider assessing any risks of offspring mutations. This study supports previous observations regarding the correlations between metabolic activity, mitochondrial distribution, and developmental competence during oocyte maturation. Similarly, early embryo development showed dynamic changes of NAD(P)H as previously reported by advanced technologies in accordance with embryonic developmental stages. Future investigations could use this approach to further develop safe, non-invasive measurements of metabolic activity using advanced novel microscopy systems and/or novel technologies to assess early development of oocytes and embryos.

## Supplementary Material

deaf196_Supplementary_Data_File_S1

deaf196_Supplementary_Figure_S1

deaf196_Supplementary_Figure_S2

deaf196_Supplementary_Table_S1

deaf196_Supplementary_Table_S2

deaf196_Supplementary_Table_S3

deaf196_Supplementary_video1

## Data Availability

The data behind this manuscript could be provided under reasonable request.
