# Supplementary material for: Investigating metabolic activity during oocyte and early embryo development through label-free metabolic imaging: a systematic approach for timelapse applications
Source: Hum Reprod. 2025 Nov 6;40(12):2272–85. doi: 10.1093/humrep/deaf196 (PMC12835920; doi:10.1093/humrep/deaf196)
Supplement: deaf196_Supplementary_Data_File_S1 [file deaf196_supplementary_data_file_s1.pdf]

Human Reproduction, 2025, 1–1
https://doi.org/10.1093/humrep/deaf196
Supplementary Data

Supplementary Data File S1. Example of calculation of energy doses for non-invasive metabolic imaging

Example of parameters in Confocal Olympus FV1200:

1. NAD(P)H: 405 nm laser power 56 mW (3–5%), scan speed 2 μm/pixel, pinhole size 800 μm, and PMT gain 960 V.
2. FAD: 473 nm laser power 17 mW (1–2%), scan speed 2 μm/pixel, pinhole size 800 μm and PMT gain 800 V.

In order to determine the optimal dose of light across the entire experiments, the following formulas were applied:

$$A_{lp} = 1/4\pi\ (D_{ph})^2 = 1/4\pi\ (8\times10^{-4}m)^2 = 50.26\times10^{-8}m^2$$

where A_{lp} and D_{ph} are area of laser spot and pinhole size, respectively. Power density of the laser beam (P_d) for each biomarker is calculated as follows:

$$P_{d,N,max} = Laser\ Power/A_{lp} = 56\ mW/50.26\times10^{-8}m^2 = 11.14\ w/cm^2$$

$$P_{d,F,max} = Laser\ Power/A_{lp} = 17\ mW/50.26\times10^{-8}m^2 = 3.38\ w/cm^2$$

Considering the maximum laser power used for imaging as 5% for NAD(P)H and 2% for FAD, the delivered power densities ($P_{d,\ N}$ *and* $P_{d,\ F}$) to the exposure area are:

$$P_{d,N} = 5\%\times11.14\ w/cm^2 = 5.57\times10^{-1}w/cm^2$$

$$P_{d,F} = 2\%\times3.38\ w/cm^2 = 0.68\times10^{-1}w/cm^2$$

Therefore, the total power density ($P_{d,\ T}$) is:

$$P_{d,T} = P_{d,N} + P_{d,F} = 6.25\times10^{-1}w/cm^2$$

Conservatively considering the area of an embryo (up to blastocyst stage) as 100 μm, the delivered power density to the imaged embryos is proportional to the laser exposure area and can be calculated as follows:

$$P_{d,T,e} = P_{d,T}x\ (D_e/D_{ph})^2 = 6.25\times10^{-1}w/cm^2\times(100/800)^2 = 9.8\ mw/cm^2$$

For each sample, 30 Z-stack imaging (n_{img}) have been performed for the two biomarkers and each Z-stack scanning time (T_{scan}) was 30 s. Therefore, the total energy density delivered ($E_{d,\ T}$) to samples for each biomarker are calculated as follows:

$$E_{d,T} = (P_{d,T,e})\times n_{img}\times T_{scan} = (9.8\times10^{-3}w/cm^2)\times30\times30s = 8.79\ J/cm^2$$
