# Supplementary material for: Investigating metabolic activity during oocyte and early embryo development through label-free metabolic imaging: a systematic approach for timelapse applications
Source: Hum Reprod. 2025 Nov 6;40(12):2272–85. doi: 10.1093/humrep/deaf196 (PMC12835920; doi:10.1093/humrep/deaf196)
Supplement: deaf196_Supplementary_Figure_S1 [file deaf196_supplementary_figure_s1.pdf]

OXFORD
human reproduction
Human Reproduction, 2025, 1–1
https://doi.org/10.1093/humrep/deaf196
Supplementary Data

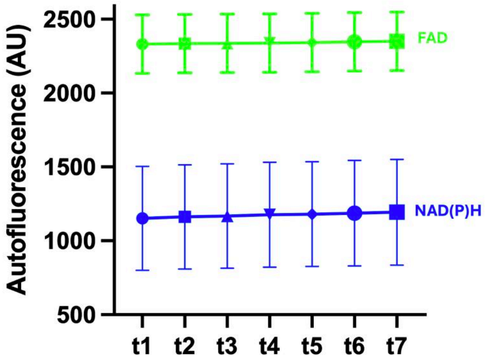

Supplementary Figure S1. Photobleaching assessment of NAD(P)H and FAD solutions overtime. NAD(P)H and FAD channel images of the same field were collected over seven timepoints every 3 min. NAD(P)H 100 μM and FAD 18 μM solutions were measured for autofluorescence using the same microscopy settings employed in live label-free metabolic imaging via confocal microscopy. Data are represented as mean ± SD.
