# Supplementary material for: Investigating metabolic activity during oocyte and early embryo development through label-free metabolic imaging: a systematic approach for timelapse applications
Source: Hum Reprod. 2025 Nov 6;40(12):2272–85. doi: 10.1093/humrep/deaf196 (PMC12835920; doi:10.1093/humrep/deaf196)
Supplement: deaf196_Supplementary_Figure_S2 [file deaf196_supplementary_figure_s2.pdf]

Human Reproduction, 2025, 1–1
https://doi.org/10.1093/humrep/deaf196
Supplementary Data

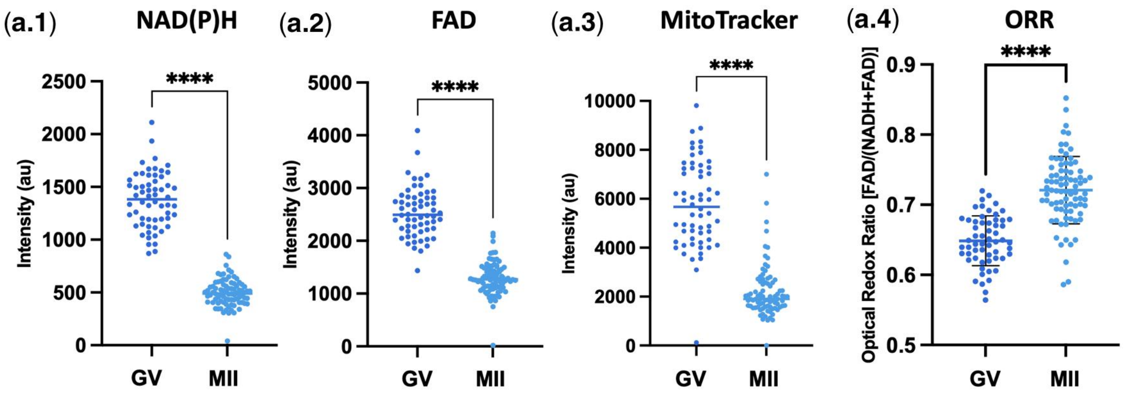

Supplementary Figure S2. NAD(P)H, FAD and Mitotracker fluorescence suggest different metabolic activity levels after *in-vitro* maturation (IVM). Germinal vesicle (GV) oocytes and oocytes that matured to metaphase II (MII) stage in the IVM experiment were stained with Mitotracker Red and metabolically imaged using confocal microscopy. GV oocytes and IVM MII oocytes showed different intensity levels using NAD(P)H, FAD, Mitotracker, and optical redox ratio (ORR). Data are represented as mean ± SEM, unpaired or paired student *t*-test was performed. ****$P < 0.001$.
