# Supplementary material for: Investigating metabolic activity during oocyte and early embryo development through label-free metabolic imaging: a systematic approach for timelapse applications
Source: Hum Reprod. 2025 Nov 6;40(12):2272–85. doi: 10.1093/humrep/deaf196 (PMC12835920; doi:10.1093/humrep/deaf196)
Supplement: deaf196_Supplementary_Table_S1 [file deaf196_supplementary_table_s1.pdf]

OXFORD human reproduction

Human Reproduction, 2025, 1–1
https://doi.org/10.1093/humrep/deaf196
Supplementary Data

Supplementary Table S1. NAD(P)H intensity levels in oocytes according to morphological status during the IVM process.

Timepoint	(A) Denuded (n)	(B) COC (n)	(C) Incomplete Development (n)	(D) Degenerated (n)	Adjusted p value	Comparison
0 hr	421.6 ± 20.94 (12)	475.6 ± 39.69 (20)	427.1 ± 43.16 (12)	660.7 ± 38.17 (11)	0.004	A–D
					0.0203	B–D
					0.0048	C–D
3 h	508.1 ± 46.56 (12)	260.5 ± 22.63 (16)	420.7 ± 39.92 (12)	256.3 ± 49.18 (12)	0.0013	A–B
					0.0424	C–D
					0.0013	A–D
6 h	342 ± 27.85 (12)	196.7 ± 18.34 (15)	252.9 ± 35.77 (11)	237.7 ± 52.33 (9)	0.0069	A–B
9 h	284.2 ± 30.63 (11)	125.0 ± 17.49 (13)	231.3 ± 28.64 (10)	201.5 ± 55.39 (7)	0.0021	A–B
12 h	212.6 ± 22.70 (11)	89.84 ± 18.66 (11)	169.4 ± 26.07 (9)	140.7 ± 53.62 (7)	0.0081	A–B
15 h	263.3 ± 14.30 (11)	109.0 ± 18.71 (14)	186.0 ± 25.57 (9)	105.8 ± 26.03 (7)	0.0001	A–B
					0.0017	A–D
18 h	200.9 ± 15.97 (11)	97.83 ± 18.00(14)	154.5 ± 22.75 (8)	82.14 ± 21.68 (5)	0.0092	A–B
					0.0184	A–D

Denude oocytes refers to oocytes without cumulus cells at the time of collection and start of IVM process; COC, cumulus oocyte complex that were cultured as COC during IVM processes; Incomplete development: oocytes that stopped development at any stage before meiosis II; Degenerated: oocytes that showed degeneration at any stage of development during IVM process; N, number of oocytes analysed. Values represent mean ± (SEM).
ANOVA test with Kruskal–Wallis for multi-comparison were applied.
