# Supplementary material for: Investigating metabolic activity during oocyte and early embryo development through label-free metabolic imaging: a systematic approach for timelapse applications
Source: Hum Reprod. 2025 Nov 6;40(12):2272–85. doi: 10.1093/humrep/deaf196 (PMC12835920; doi:10.1093/humrep/deaf196)
Supplement: deaf196_Supplementary_Table_S2 [file deaf196_supplementary_table_s2.pdf]

OXFORD
human reproduction
Human Reproduction, 2025, 1–1
https://doi.org/10.1093/humrep/deaf196
Supplementary Data

Supplementary Table S2. FAD intensity levels in oocytes according to morphological status during the IVM process.

Timepoint	(A) Denuded (n)	(B) COC (n)	(C) Incomplete development (n)	(D) Degenerated (n)	Adjusted *P* value	Comparison
0 h	1225 ± 64.28 (13)	947.5 ± 44.17 (20)	1097 ± 64.23 (12)	1270 ± 99.77 (12)	0.0147	A–B
					0.0122	B–D
3 h	1050 ± 62.43 (13)	650 ± 52.08 (20)	935.4 ± 46.19 (12)	909.7 ± 71.53 (12)	0.0002	A–B
					0.0091	B–C
6 h	874.7 ± 48.72 (13)	591.2 ± 52.34 (18)	747.1 ± 55.03 (12)	737.2 ± 108.0 (12)	0.0052	A–B
9 h	772.1 ± 72.42 (13)	524.7 ± 46.30 (17)	662.4 ± 66.70 (12)	587.3 ± 126.1 (12)	0.0235	A–B
12 h	664.8 ± 61.79 (13)	481.4 ± 34.24 (16)	628.3 ± 33.18 (11)	767.6 ± 108.5 (11)	0.0283	A–B
					0.0047	B–D
15 hrs	682.1 ± 61.33 (13)	501.5 ± 31.82 (16)	600.1 ± 38.68 (11)	559.8 ± 105.8 (11)	0.0199	A–B
18 hrs	672.9 ± 30.70 (13)	479.5 ± 33.84 (16)	515.9 ± 42.69 (11)	621.0 ± 112.7 (9)	0.0434	A–B

Denude oocytes refers to oocytes without cumulus cells at the time of collection and start of IVM process; COC, cumulus oocyte complex that were cultured as COC during IVM processes; Incomplete development: oocytes that stopped development at any stage before meiosis II; Degenerated: oocytes that showed degeneration at any stage of development during IVM process; N, number of oocytes analysed. Values represent mean ± (SEM).
ANOVA test with Bonferroni test for multi-comparison were applied.
