# Supplementary material for: Investigating metabolic activity during oocyte and early embryo development through label-free metabolic imaging: a systematic approach for timelapse applications
Source: Hum Reprod. 2025 Nov 6;40(12):2272–85. doi: 10.1093/humrep/deaf196 (PMC12835920; doi:10.1093/humrep/deaf196)
Supplement: deaf196_Supplementary_Table_S3 [file deaf196_supplementary_table_s3.pdf]

Human Reproduction, 2025, 1–1
https://doi.org/10.1093/humrep/deaf196
Supplementary Data

Supplementary Table S3. Optical Redox ratio levels in oocytes according to morphological status during the IVM process.

Timepoint	(A) Denuded (n)	(B) COC (n)	(C) Incomplete development (n)	(D) Degenerated (n)	Adjusted *P* value	Comparison
0 h	0.7474 ± 0.007221 (12)	0.6701 ± 0.02249 (20)	0.7212 ± 0.02527 (12)	0.6514 ± 0.02370 (11)	0.0452	A–D
3 h	0.6848 ± 0.01377 (12)	0.7489 ± 0.01436 (16)	0.6944 ± 0.01603 (12)	0.7973 ± 0.01895 (12)	0.0008	A–D
					0.0024	C–D
6 h	0.7259 ± 0.008601 (12)	0.7793 ± 0.01220 (15)	0.7667 ± 0.02362 (11)	0.8061 ± 0.03289 (9)	0.0209	A–B
9 h	0.7526 ± 0.01098 (11)	0.8287 ± 0.01725 (13)	0.7664 ± 0.01404 (10)	0.8301 ± 0.03680 (7)	0.0166	A–B
12 h	0.7793 ± 0.01442 (11)	0.8638 ± 0.02023 (11)	0.7988 ± 0.01618 (9)	0.8758 ± 0.03880 (7)	0.0487	A–B
15 h	0.7410 ± 0.009582 (11)	0.8373 ± 0.02134 (14)	0.7778 ± 0.01800 (9)	0.8803 ± 0.02786 (7)	0.0133	A–B
					0.0017	A–D
18 hrs	0.7766 ± 0.009331 (11)	0.8498 ± 0.01892 (14)	0.7908 ± 0.01557 (8)	0.8913 ± 0.03245 (5)	0.0319	A–D

Denude oocytes refers to oocytes without cumulus cells at the time of collection and start of IVM process; COC: cumulus oocyte complex that were cultured as COC during IVM processes; Incomplete development: oocytes that stopped development at any stage before meiosis II; Degenerated: oocytes that showed degeneration at any stage of development during IVM process; N, number of oocytes analysed. Values represent mean ± (SEM).
ANOVA test with Bonferroni test for multi-comparison were applied.

© The Author(s) 2025. Published by Oxford University Press on behalf of European Society of Human Reproduction and Embryology.
This is an Open Access article distributed under the terms of the Creative Commons Attribution License (https://creativecommons.org/licenses/by/4.0/), which permits unrestricted reuse, distribution, and reproduction in any medium, provided the original work is properly cited.
Human Reproduction, 2025, 00, 1–21
https://doi.org/10.1093/humrep/deaf196
Original Article
